# Antidepressant and antipsychotic side-effects and personalised prescribing: a systematic review and digital tool development

**DOI:** 10.1016/S2215-0366(23)00262-6

**Published:** 2023-11

**Authors:** Toby Pillinger, Oliver D Howes, Christoph U Correll, Stefan Leucht, Maximilian Huhn, Johannes Schneider-Thoma, Fiona Gaughran, Sameer Jauhar, Philip K McGuire, David M Taylor, Allan H Young, Robert A McCutcheon

**Affiliations:** a Department of Psychosis Studies, Institute of Psychiatry, Psychology and Neuroscience, King's College London, London, UK; b Department of Psychological Medicine, Institute of Psychiatry, Psychology and Neuroscience, King's College London, London, UK; c Psychiatric Imaging Group, MRC London Institute of Medical Sciences, Imperial College London, London, UK; d Department of Psychiatry, Zucker Hillside Hospital, Northwell Health, New York, NY, USA; e Department of Psychiatry and Molecular Medicine, Donald and Barbara Zucker School of Medicine at Hofstra/Northwell, Hempstead, NY, USA; f Department of Child and Adolescent Psychiatry, Charité – Universitätsmedizin Berlin, Berlin, Germany; g Department of Psychiatry and Psychotherapy, School of Medicine, Technical University of Munich, Munich, Germany; h South London and Maudsley NHS Foundation Trust, London, UK; i Department of Psychiatry, University of Oxford, Warneford Hospital, Oxford, UK; j Pharmacy Department, Maudsley Hospital, South London and Maudsley NHS Foundation Trust, London, UK; k Institute of Pharmaceutical Science, King's College London, London, UK; l South London and Maudsley NHS Foundation Trust, Bethlem Royal Hospital, Beckenham, UK; m Oxford Health NHS Foundation Trust, Oxford, UK

## Abstract

**Background:**

Side-effects of psychiatric medication impair quality of life and functioning. Furthermore, they contribute to morbidity, mortality, stigma, and poor treatment concordance resulting in relapse of psychiatric illness. Guidelines recommend discussing side-effects with patients when making treatment decisions, but a synthesis of antidepressant and antipsychotic side-effects to guide this process is missing, and considering all side-effects is a complex, multidimensional process. We aimed to create comprehensive databases of antipsychotic and antidepressant side-effects, and a digital tool to support database navigation.

**Methods:**

To create the databases, we did an umbrella review of Embase, PsycINFO, and MEDLINE from database inception to June 26, 2023. We included meta-analyses of randomised controlled trials examining antipsychotic monotherapy in the treatment of schizophrenia or antidepressant monotherapy in the treatment of major depressive disorder. We included meta-analyses in adults (aged ≥18 years) that assessed drugs with a common comparator. The search was complemented by a review of national and international guidelines and consensus statements for the treatment of major depressive disorder and schizophrenia in adults. Effect sizes for antipsychotic and antidepressant side-effects were extracted from meta-analyses examining the largest number of drugs. In cases of incomplete meta-analytic coverage, data were imputed on the basis of guideline-derived ordinal rankings or, if imputation was not possible, ordinal scores were extracted. Both meta-analytic and ordinal outcomes were normalised to provide values between 0 and 1. We then constructed a digital tool, the Psymatik Treatment Optimizer, to combine the side-effect databases with side-effect concerns of an individual user, to enable users to select side-effects of concern and the relative degree of concern for each side-effect. Concern weightings and the side-effect databases are synthesised via a multicriteria decision analysis method (technique for order of preference by similarity to ideal situation, or TOPSIS).

**Findings:**

Of 3724 citations, 14 articles containing 68 meta-analyses of individual side-effects met inclusion criteria. After review of 19 guidelines, seven provided ordinal data. Antipsychotic data were extracted from five studies (11 meta-analyses, n=65 594 patients) and four guidelines, and antidepressant data were extracted from three guidelines. The resultant databases included data on 32 antipsychotics (14 side-effects) and 37 antidepressants (nine side-effects). The databases highlighted the clinical dilemma associated with balancing side-effects, with avoidance of one side-effect (eg, weight gain for antipsychotics) increasing the risk of others (eg, akathisia). To aid with this dilemma, the Psymatik Treatment Optimizer synthesises the side-effect databases with individual user-defined concern weights. After computing up to 5851 pairwise comparisons for antidepressants and 5142 pairwise comparisons for antipsychotics, Psymatik ranks treatments in order of preference for the individual user, with the output presented in a heatmap.

**Interpretation:**

By facilitating collaborative, personalised, and evidence-based prescribing decisions, the side-effect databases and digital application supports care delivery that is consistent with international regulatory guidance for the treatment of schizophrenia and depression, and it therefore has promise for informing psychiatric practice and improving outcomes.

**Funding:**

National Institute for Health and Care Research, Maudsley Charity, Wellcome Trust, Medical Research Council.

## Introduction

In high-income countries, up to 17% of adults are prescribed antidepressants,^1^, ^2^, ^3^ and up to 2% are prescribed antipsychotics.^4^, ^5^ Rates of prescribing of antidepressants and antipsychotics are increasing year on year.^6^ Although antipsychotics and antidepressants are effective treatments in some patients,^7^, ^8^ approximately 75% of patients experience side-effects.^9^, ^10^ These side-effects are diverse^11^ and can impair quality of life and functioning,^12^ contribute to morbidity and mortality rates,^13^ create stigma,^14^ and result in poor medication concordance and thus increased risk of relapse of psychiatric illness.^15^ Indeed, side-effects are factors that people with depression and schizophrenia primarily consider when making prescription decisions,^16^, ^17^ and concerns about side-effects represent a barrier to treatment of mental illness.^18^ National and professional bodies recommend that side-effect discussions are central to antipsychotic and antidepressant prescribing decisions.^19^, ^20^, ^21^, ^22^ However, simultaneously considering the relative side-effect profile for every available antidepressant and antipsychotic is a complex, multidimensional process. For example, a discussion regarding which of 20 antidepressants to select on the basis of the relative risk of ten side-effects involves 1900 pairwise comparisons; 10 s spent considering each comparison would require more than 5 h of discussion. This process is further complicated by the fact that avoidance of some side-effects will be important to one patient but not to another, meaning that at an individual level, greater consideration needs to be given to avoiding specific side-effects over others. Thus, under current practice, engaging in comprehensive, evidence-based, and personalised psychotropic prescribing is an almost insurmountable task. This directly affects clinical care, with inadequate presentation of side-effect-related information identified by both patients and professional bodies as being a hindrance to the decision-making process for psychiatric medications.^2^, ^16^

**Research in context**

**Evidence before this study**

Although clinical guidelines stipulate the need for discussion between clinician and patient about antidepressant and antipsychotic side-effects before making prescription decisions, they do not specify how this complex, multidimensional process should be done. We searched PubMed for studies that provide an evidence-based synthesis of the relative risk or magnitude of antidepressant or antipsychotic side-effects using data from randomised controlled trials, with the search terms “(antipsychotic OR antidepressant) AND side effect”, filtering for systematic review. We then searched PubMed from database inception up to Jan 28, 2023, with no language restrictions, for studies describing a digital tool that facilitates antidepressant or antipsychotic prescriptions in people with depression or schizophrenia by considering all side-effects of all treatments while also incorporating individual patient concerns, with the search terms “(antipsychotic OR antidepressant OR depression OR schizophrenia) AND side effect AND digital tool)”. Of the 737 studies retrieved, 14 provided meta-analytic data for antipsychotic or antidepressant side-effects. However, these studies were limited by either focussing on a restricted range of side-effects or a limited number of drugs. Thus, no studies were identified that have provided clinicians with a comprehensive, evidence-based synthesis of antidepressant or antipsychotic side-effects. Furthermore, no studies were identified describing a digital application to facilitate antidepressant or antipsychotic prescription decision-making on the basis of side-effects and patient concerns.

**Added value of this study**

This study synthesises multiple high-quality resources, and, to our knowledge, provides the most comprehensive antidepressant and antipsychotic side-effect database to date. In theory, the database could be used in isolation to inform prescribing discussions between patients and clinicians. However, the clinician and patient would still be faced with the complexity of simultaneously making hundreds, if not thousands, of drug–side-effect pairwise comparisons. To address this challenge, we developed a digital tool that supports navigation of the side-effect database while incorporating user concerns.

**Implications of all the available evidence**

By facilitating multidimensional, collaborative, and personalised prescribing decisions, the present work addresses a fundamental shortcoming of modern psychiatric practice, and has immediate implications for the day-to-day clinical care of people with schizophrenia and depression.

Intolerable side-effects, alongside inadequate treatment response, contribute to the switching of psychiatric medications.^23^, ^24^ Antidepressant switching occurs in approximately 9% of patients with a new episode of depression,^25^ and antipsychotic switching or premature discontinuation occurs in 73% of patients with first-episode psychosis.^26^ Switching treatment is associated with health-economic and societal costs. For example, annual health-care costs in the USA are US$3000 higher in individuals who switch antidepressants than in those who do not.^27^ Furthermore, patients who switch antidepressants have a 70% reduction in workplace productivity and twice as many days absent from work versus individuals who do not switch medication.^27^ Thus, initiatives that aim to reduce the risk of medication switching, such as personalised prescribing, should improve not only clinical outcomes but also health-economic and functional outcomes.

In the past decade, a large amount of research has ranked psychiatric treatments on the basis of efficacy and risk of side-effects.^7^, ^8^, ^28^ This research is often in the form of network meta-analyses, an approach that simultaneously compares effects between multiple interventions. The results of network meta-analyses have had immediate clinical impact. For example, a network meta-analysis that ranked antipsychotics according to degree of metabolic dysregulation^28^ formed the basis of a metabolically minded prescribing guidance.^29^ However, side-effects of psychiatric treatments are broader than just metabolic disturbance, and an optimal approach should consider a wider range of side-effects. To our knowledge, a comprehensive synthesis of meta-analyses examining side-effects of antidepressants and antipsychotics in the adult population has not been done. Furthermore, to optimise prescribing, it is necessary to address the challenges of multidimensional decision making (ie, simultaneously comparing multiple side-effects for multiple treatments) while also incorporating side-effect data and preferences of the patient. To address these issues, we did an umbrella review of previous meta-analyses that compared and ranked antidepressants or antipsychotics on the basis of side-effect burden in adults, and we extracted these data to construct side-effect databases (one for antipsychotics and one for antidepressants). We then designed a digital application that combines the side-effect databases with user preference, to facilitate evidence-based and personalised prescribing decisions.

## Methods

### Search strategy and selection criteria

**Figure 1:**
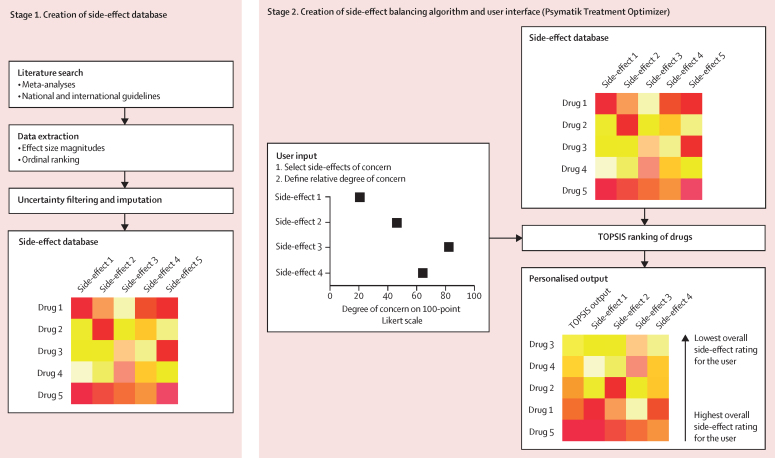
Methods summary First, evidence-based side-effect databases were created, one each for antipsychotics and antidepressants. Second, a digital tool was devised that allows the user to input side-effects of concern and the relative degree of concern for each side-effect. The input data are synthesised with the side-effect database using TOPSIS, the output of which ranks treatments from best to worst. Side-effect 1 represents the first side-effect of interest (eg, sedation), side-effect 2 represents the second side-effect of interest (eg, weight gain), and so on. Drug 1 represents the first antipsychotic or antidepressant of interest, drug 2 represents the second antipsychotic or antidepressant of interest, and so on. TOPIS=technique for order of preference by similarity to ideal situation.

The study had two methodological stages (figure 1). For the first stage of creating side-effect databases, we did a systematic umbrella review of published meta-analyses following a prespecified protocol, registered with PROSPERO (CRD42022372142; appendix pp 2–5). Two authors (TP and RAMcC) separately searched Embase, PsycINFO, and MEDLINE from database inception up to June 26, 2023, for meta-analyses that ranked antidepressants when used as monotherapy by individuals with major depressive disorder, or antipsychotics when used as monotherapy by individuals with schizophrenia, on the basis of relative magnitude or risk of side-effects. The search results were cross-checked with differences resolved by discussion between the two authors. To allow broad drug comparisons, we focused on side-effects that are common among antipsychotics or antidepressants, as defined in clinical guideline documents.^29^, ^30^ As such, we did not include side-effects considered to be largely clozapine specific (eg, myocarditis and agranulocytosis).^30^ The search terms used were: (antidepressant OR antipsychotic) AND (extrapyramidal OR parkinsonism OR dyskinesia OR dystonia OR akathisia OR prolactin OR headache OR agitation OR insomnia OR cholinergic OR gastrointestinal OR constipation OR nausea OR arrythmia OR QTc OR hypotension OR hypertension OR weight OR glucose OR lipid OR cholesterol OR triglyceride OR sedation OR natraemia OR natremia OR sodium OR thromboembolism OR sexual) AND meta-analysis. To reduce heterogeneity, we only included meta-analyses of randomised controlled trials examining antidepressant or antipsychotic monotherapy in the treatment of depression or schizophrenia, respectively, and drugs assessed within the meta-analysis needed a common comparator. We also only included studies on adults aged 18 years or older. In recognition that some side-effects (eg, venous thromboembolism and tardive dyskinesia) might occur after longer-term treatment, we included meta-analyses of trials examining both acute and maintenance treatment. Only studies written in English were selected. Summary estimates were sought and, if data were missing, study authors were contacted. In anticipation of inadequate meta-analytic coverage of some side-effects, we aimed to supplement results with ordinal ranking data from guidelines for the treatment of major depressive disorder and schizophrenia. As such, in parallel to the meta-analytic review, we also reviewed national and international guideline documents and similar sources (eg, consensus statements and clinical guideline resources) for the management of schizophrenia or major depressive disorder in adult populations (age ≥18 years); these documents were selected from the reference lists of previous umbrella reviews in the field (appendix p 9). Guidelines were selected if they provided ordinal rankings for drug side-effects.

In recognition that patients might wish to consider both the side-effects and efficacy of a given treatment, we did a second umbrella review (not specified a priori in the protocol) of antipsychotic or antidepressant efficacy in the respective monotherapy of schizophrenia or depression, and created databases of the efficacy data. The search details for this second review are provided in the appendix (p 9), and subsequent steps for database creation were the same for efficacy data.

### Data analysis

The side-effect databases were populated by meta-analytic, ordinal, and imputed data. Any meta-analytic effect size estimates representing the risk or magnitude of side-effects (eg, relative risk, mean difference, standardised mean difference, odds ratio, or incidence rate) were extracted separately by two authors (TP and RAMcC) and cross-checked, with differences resolved by discussion between the two authors. Ordinal data from guidelines ranking drugs on the basis of the magnitude or risk of specific side-effects were also extracted. Meta-analytic data were preferred over ordinal data. When more than one meta-analysis was available for a given side-effect, we selected the meta-analysis examining the largest number of drugs. If mean difference and standardised mean difference were both provided for a given side-effect, the analysis with lower heterogeneity was selected.

The methodological quality of each meta-analysis was assessed with the Measurement Tool to Assess Systematic Reviews 2 checklist (appendix p 9); confidence in meta-analytic results was scored out of a maximum of 16. Overall confidence was rated as specifed by AMSTAR2, as high (no or one non-critical weakness); moderate (more than one non-critical weakness); low (one critical flaw with or without non-critical weaknesses); and critically low (more than one critical flaw with or without non-critical weaknesses). To assess confidence in ordinal rankings, we examined the references used as justification, and we assessed methodological quality with the Appraisal of Guidelines for Research and Evaluation II reporting checklist (appendix p 9). On this checklist, total score was standardised as a percentage of the maximum possible score of 161. We defined quality by the thresholds: ≥80%, outstanding; <80% and ≥70%, excellent; <70% and ≥60%, good; <60% and ≥50%, satisfactory; <50% and ≥40%, poor; and <40%, very poor. Thresholds were selected by consensus agreement between authors.

Both meta-analytic and ordinal outcomes were normalised (minimum–maximum scaled) to provide values between 0 and 1. These normalised scores were used as proxy effect sizes. When there was more than one meta-analysis for a given side-effect, we selected the meta-analysis examining the largest number of drugs. Thus, meta-analyses with sample overlap were excluded. When there were multiple guidelines ranking drugs for the same side-effect, a mean of normalised scores was calculated. We imputed missing effect sizes for drug–side-effect pairs on the basis of ordinal ranking scores, as follows. When there were ten or more of each type of datapoint (≥10 meta-analytic effect sizes and ≥10 ordinal ranking scores) for the same drug–side-effect pair, linear regression was used to define the relationship between ordinal ranking scores (the predictor, or independent, variable) and meta-analytic effect sizes (the predicted, or dependent, variable) for the given side-effect. This model was then used to predict missing meta-analytic effect sizes for other drugs on the basis of available ranking scores. If there were fewer than ten overlapping datapoints and imputation was not possible, ordinal scores were extracted. If multiple meta-analyses had examined the same side-effect for the same number of drugs, we selected the meta-analysis with the largest regression model coefficient of determination. The validity of some side-effect network meta-analysis results has been queried, with inclusion of small outlier studies blamed.^7^ Therefore, we excluded meta-analytic effect sizes and used imputed data when there were major uncertainty concerns. Major uncertainty for a given drug–side-effect pair was defined as an accompanying 95% CI that was at least double the mean of 95% CIs for all drugs for that side-effect. To minimise drug exclusion, meta-analytic data (direct or imputed) were only used for a given side-effect if available for at least 66% of the total number of antipsychotics and antidpepressants for which we were able to gather side-effect data. The cutoff of 66% was selected by study authors. When coverage was less than 66%, ordinal rankings were used. The resultant databases consisted of rows corresponding to drugs, and columns corresponding to side-effects, with entries corresponding to meta-analytic effect sizes, imputed meta-analytic effect sizes, or guideline-defined ordinal ranking scores. Further details are provided in the appendix (pp 9–10). Analyses were done with R (version 3.6.1).

### Side-effect balancing algorithm and user interface

For the second stage of the study (figure 1), we designed a web application, available online, using the Django tool. The application was made to be accessible by either desktop or mobile devices. On this application, two digital tools were created, one each for antipsychotics and antidepressants. The user selects side-effects of concern; they then weight the relative degree of concern for each of those side-effects by adjusting a slider between poles of “minor concern” and “major concern”. These weightings are translated into a 100-point Likert scale. For selected side-effects, the user-defined weighting and normalised effect size magnitude for each drug with complete side-effect data are synthesised via the technique for order of preference by similarity to ideal solution (TOPSIS) method.^31^ TOPSIS is a multicriteria decision analysis method that ranks alternative solutions (here, medications) by considering more than one criterion (here, side-effects). It is based on the concept that the best solution should have the shortest geometric distance from the positive ideal solution (the best values available across all criteria) and the longest geometric distance from the negative ideal solution (the worst values attainable across all criteria). The TOPSIS ranking is displayed in a heatmap, in which drugs with the lowest overall personalised side-effect scores are placed at the top. Raw efficacy data for drugs are visually presented as a separate heatmap column alongside the TOPSIS results.

### Role of the funding source

The funders of the study had no role in study design, data collection, data analysis, data interpretation, or writing of the report.

## Results

**Figure 2:**
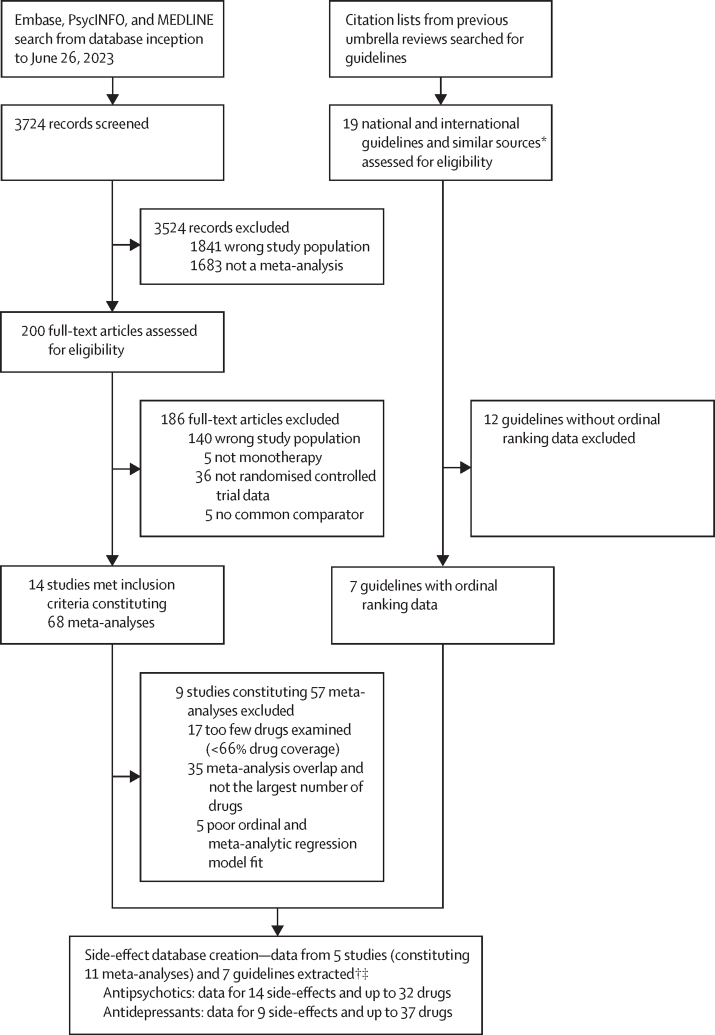
Search process *Including consensus statements and one clinical guideline resource (UpToDate). †Including the UpToDate resource. ‡For antipsychotics, data were from five studies (constituting 11 meta-analyses) and four guideline documents; for antidepressants, data were from three guideline documents.

**Figure 3:**
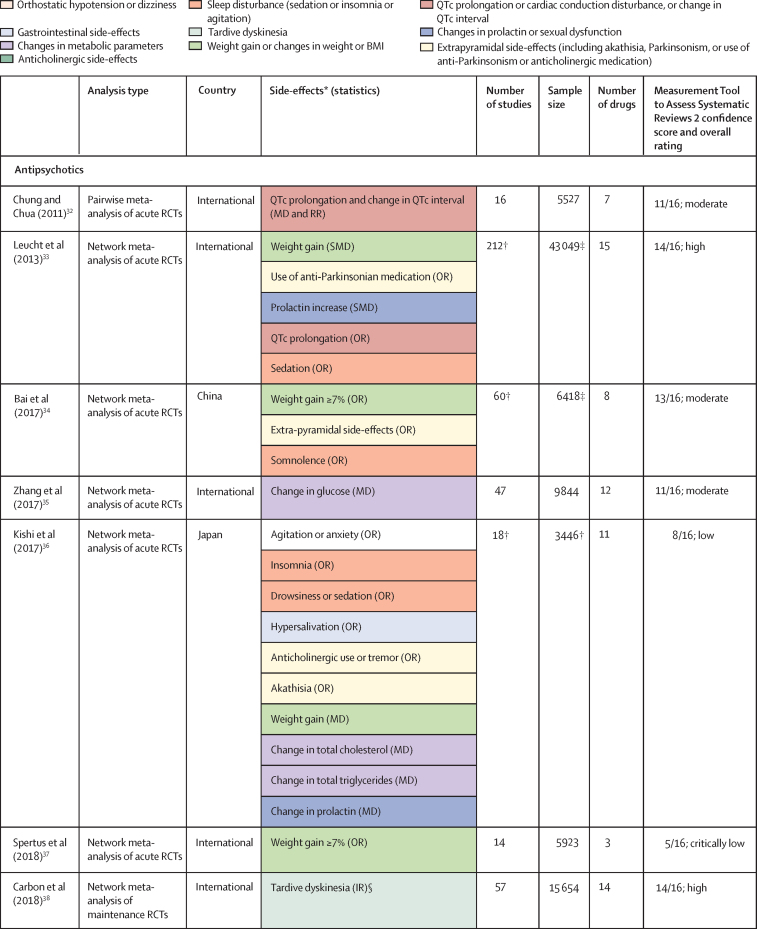

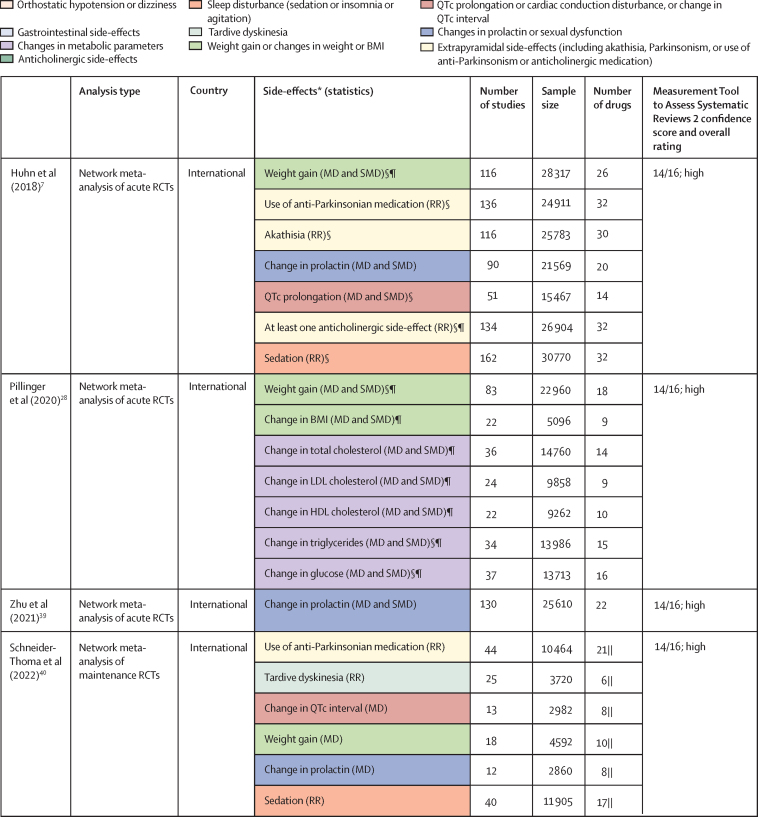

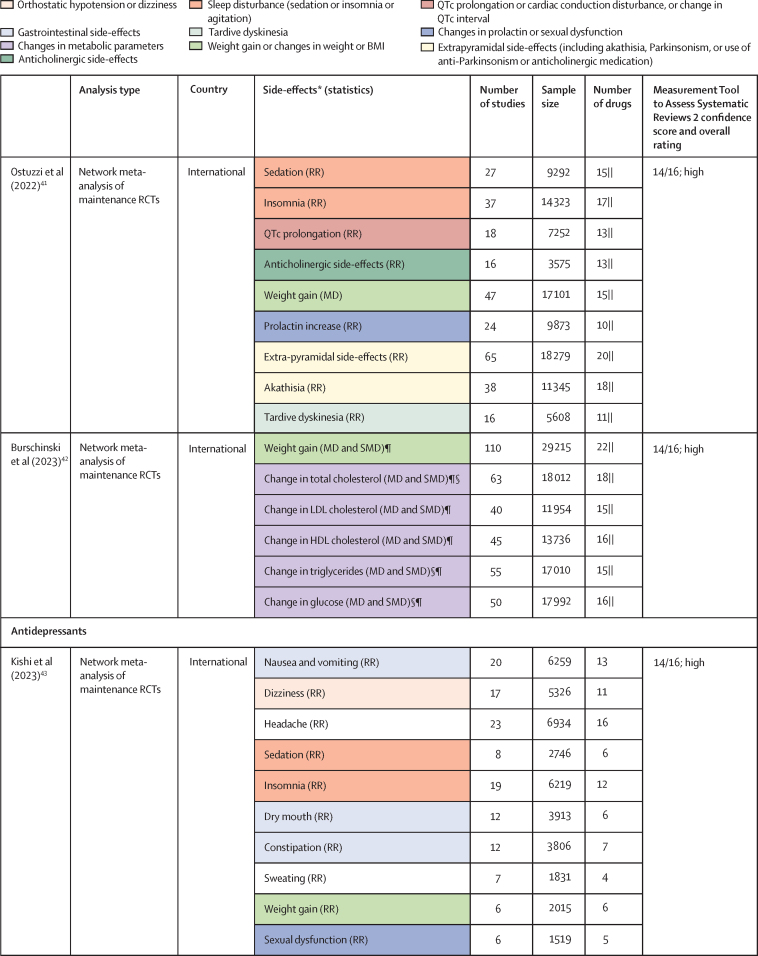
Meta-analyses included in umbrella review Colour coding shows groups of similar side-effects for those with more than one mention. HDL=high-density lipoprotein. IR=incidence rate. LDL=low-density lipoprotein. MD=mean difference. OR=odds ratio. QTc=corrected QT interval. RCT=randomised controlled trial. RR=relative risk. SMD=standardised mean difference. *Unless specified, no defined cutoff for change in parameter provided. †This number refers to the overall number of studies included in the network meta-analysis; study numbers for individual side-effect analyses (eg, weight gain or prolactin increase) were not provided. ‡This number refers to the overall sample size included in the network meta-analysis; sample sizes for individual side-effect analyses were not provided. §Selected for data extraction for the side-effect database. ¶Unpublished data provided by authors. ||Oral and injectable formulations of the same drug counted as a single drug.

**Figure 4:**
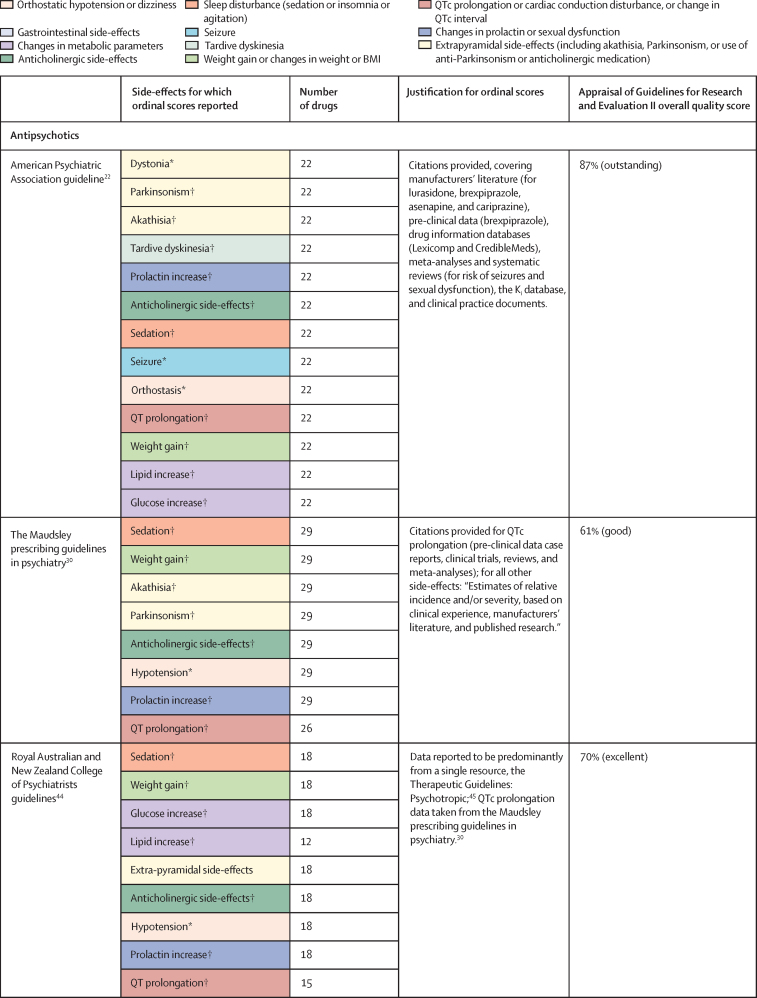

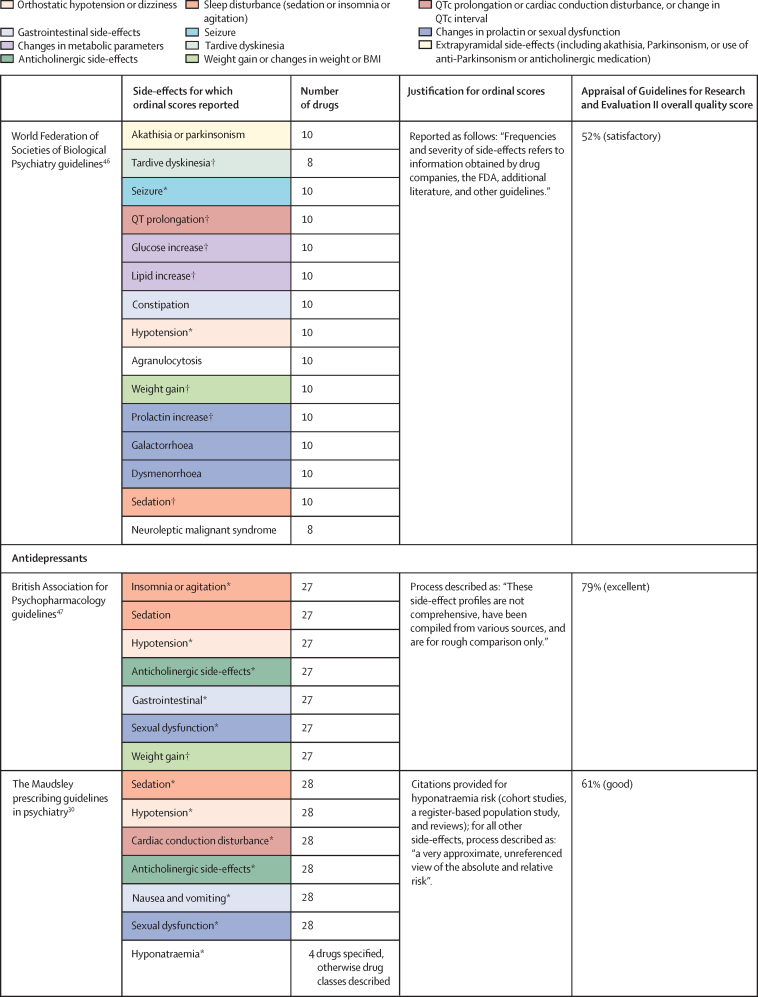

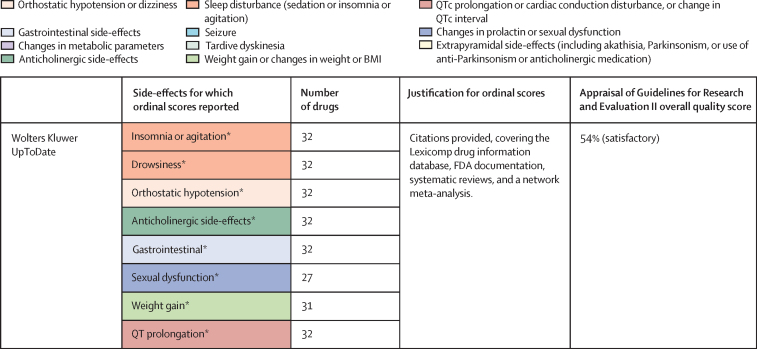
National and international guidelines providing rankings of antipsychotic or antidepressant side-effects Colour coding shows groups of similar side-effects for those with more than one mention. See online for the UpToDate clinical resource. QTc=corrected QT interval. FDA=US Food and Drug Administration. *Selected for data extraction for the side-effect database. †Used for data imputation.

Of 3724 citations retrieved, 14 studies constituting 68 meta-analyses of individual side-effects met inclusion criteria (Figure 2, Figure 3). Of 19 guideline documents and similar sources that were screened, six national or international guidelines and the international UpToDate clinical resource (hereafter collectively referred to as guidelines) provided ordinal ranking data (figure 4).

**Figure 5:**
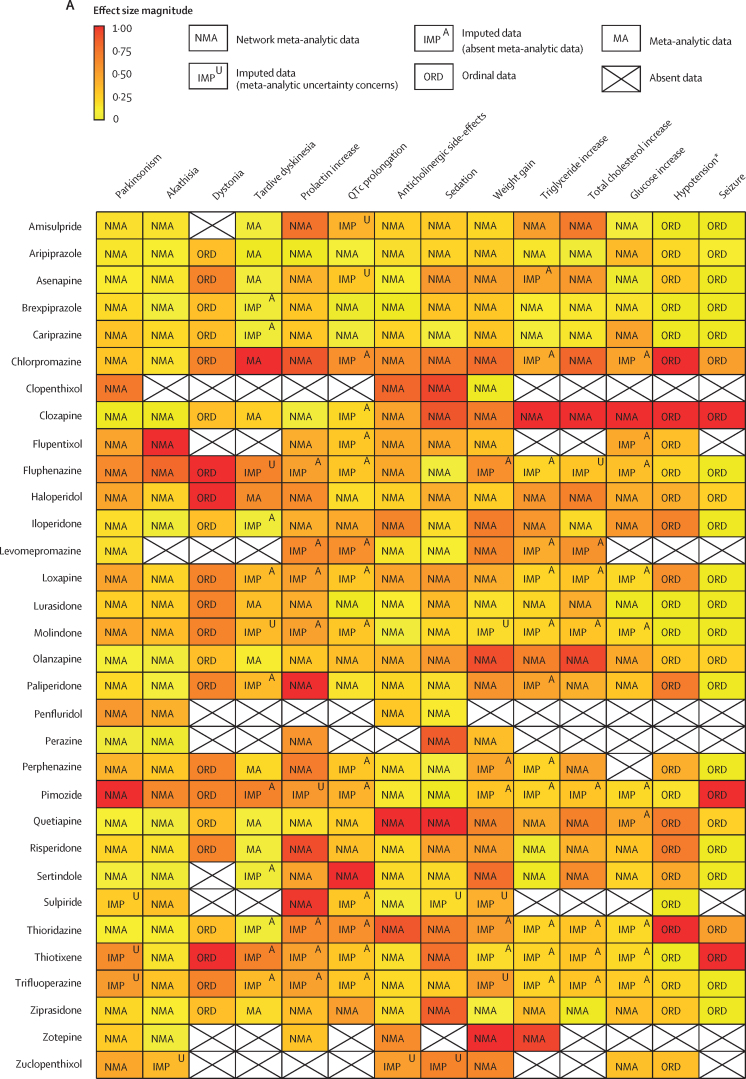

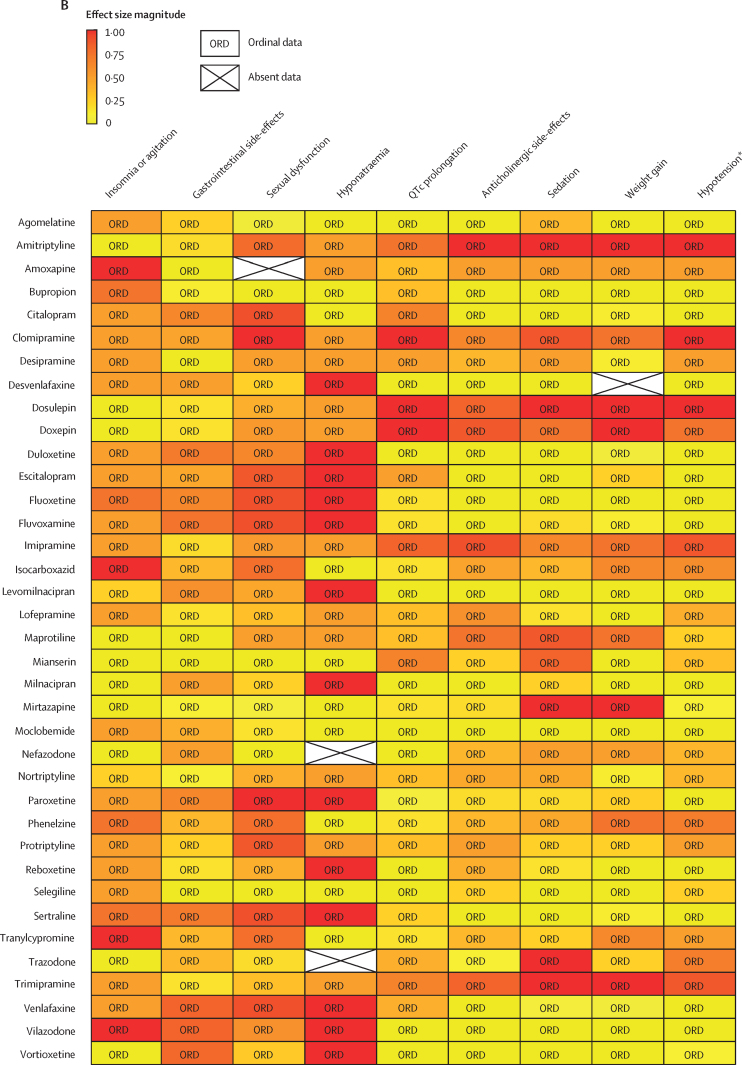
Heatmaps representing the Psymatik antipsychotic and antidepressant side-effect databases (A) Antipsychotic side-effect database. (B) Antidepressant side-effect database. Effect size magnitudes are normalised such that all values are between 0 and 1 inclusive; the higher the effect size, the greater the risk or magnitude of the side-effect for that drug. Effect sizes represent either the risk of a given side-effect happening or the magnitude of a given side-effect. Data are a combination of network meta-analytic, pairwise meta-analytic, imputed, and ordinal data. Data were imputed either because of uncertainty concerns regarding meta-analytic outcomes or because of absent meta-analytic data. QTc=corrected QT interval. *Including orthostatic hypotension.

For antipsychotics, meta-analytic data were available from 13 studies, containing 58 meta-analyses addressing 18 side-effects (figure 3). We assumed that use of antiparkinsonian medication described emergence of Parkinsonism and therefore grouped this side-effect description with tremor. Furthermore, we assumed that somnolence, sedation, and drowsiness described the same side-effect. Ordinal data were identified from four national or international guidelines for 19 side-effects (figure 4). We assumed that akathisa or Parkinsonism and extrapyramidal side-effects described the same group of side-effects. Furthermore, we assumed that orthostasis and hypotension broadly described the same side-effect. Five studies remained after selecting those with the largest numbers of drugs or best imputation model fit and excluding those with insufficient drug numbers (appendix pp 11–12).^7^, ^28^, ^38^, ^39^, ^42^ These studies constituted 11 side-effect meta-analyses for up to 32 antipsychotics and an overall sample size of 65 594 patients from at least 38 countries. These data were complemented by ordinal ranking data from four guidelines for three other side-effects. The 14 side-effects were: Parkinsonism, akathisia, dystonia, tardive dyskinesia, change in prolactin, corrected QT (QTc) interval prolongation, anticholinergic side-effects, sedation, weight gain, change in triglycerides, change in total cholesterol, change in glucose, hypotension (including orthostasis), and seizure. Imputation was performed for all 11 side-effects with meta-analytic data (imputation model R^2^ range 0·24–0·70, appendix p 12). Of 448 potential drug–side-effect pair datapoints, 231 (52%) were extracted directly from meta-analyses, 80 (18%) were imputed, and 73 (16%) were ordinal data; data were not available for 64 (14%) drug–side-effect pairs (figure 5A; appendix p 13). Confidence ratings for meta-analyses ranged from critically low to high, although all meta-analyses selected for data extraction were rated high (figure 3). Of the four guidelines from which ordinal data were extracted, two provided comprehensive references to justify results (American Psychiatric Association guidelines^22^ and Royal Australian and New Zealand College of Psychiatrists guidelines^44^), one provided a partial list of references (Maudsley prescribing guidelines^30^), and one did not provide references (World Federation of Societies of Biological Psychiatry guidelines^46^). Methodological quality of guidelines ranged from satisfactory to outstanding (figure 4, appendix p 14). Across meta-analyses and guidelines, we observed consistent grouping of antipsychotics on the basis of contrasting side-effect profiles (figure 5A). For example, partial agonists (aripiprazole, brexpiprazole, and cariprazine), lurasidone, and ziprasidone were low-risk drugs for weight gain and metabolic dysregulation. However, this same group tended to confer an increased risk of akathisia, although not to the extent of older drugs such as haloperidol, flupentixol, zuclopenthixol, fluphenazine, and pimozide. These older drugs also increased the risk of Parkinsonism and hyperprolactinaemia; however, they were observed to be more benign in terms of weight gain. First-generation antipsychotics (eg, chlorpromazine, flupentixol, and haloperidol) and the second-generation drugs amisulpride, risperidone, and paliperidone conferred increased risk of hyperprolactinaemia. By contrast, the second-generation drugs olanzapine, clozapine, quetiapine, and zotepine were higher risk drugs for weight gain, metabolic dysregulation, sedation, and anticholinergic side-effects. However, this same group tended to confer low risk for movement side-effects and hyperprolactinaemia.

For antidepressants, a single study examining maintenance antidepressant therapy met inclusion criteria. Although this included ten meta-analyses, no side-effect had meta-analytic data available for at least 66% of the drugs for which we gathered data. For two groups of side-effects (insomnia or agitation; and nausea and vomiting), there were sufficient overlapping ordinal and meta-analytic datapoints to allow imputation modelling. However, for both groups, we observed a negative relationship between ordinal and meta-analytic data (appendix p 14). Owing to concerns regarding the validity of these models, we did not proceed to imputation. Instead, ordinal side-effect data were extracted from three national or international guidelines. To synthesise ordinal rankings across guidelines, we assumed the following descriptions described broadly similar side-effects: gastrointestinal and nausea and vomiting; QTc prolongation and cardiac conduction disturbance; and drowsiness and sedation. We extracted data for nine side-effects and up to 37 drugs (figure 4, figure 5B), as follows: QTc prolongation, hypotension (including orthostasis), insomnia or agitation, sedation, anticholinergic side-effects, gastrointestinal side-effects, sexual dysfunction, weight-gain, and hyponatraemia. Data were missing for four (1%) of 333 drug–side-effect pairs. Of the three guidelines from which ordinal data were extracted, one provided comprehensive references to justify results (UpToDate), one provided a partial list of references (Maudsley prescribing guidelines^30^), and one did not provide references (British Association for Psychopharmacology guidelines^47^). Methodological quality of guidelines ranged from satisfactory to excellent (figure 4, appendix p 14). Side-effect patterns were evident across antidepressant classes, although we noted unique side-effect signatures for particular drugs within classes (figure 5B). For example, selective serotonin reuptake inhibitors (eg, fluoxetine and sertraline) were broadly associated with gastrointestinal and sexual side-effects and high risk of hyponatraemia, but with lower risk of anticholinergic and sedating effects. However, the risk of QTc prolongation varied among selective serotonin reuptake inhibitors, with citalopram conferring the highest risk and sertraline the lowest. Tricyclic antidepressants (eg, amitriptyline and trimipramine) broadly conferred increased risk of weight gain, sedation, orthostatic hypotension, sexual dysfunction, and anticholinergic side-effects; however, risk of insomnia or agitation and gastrointestinal effects was lower. Risk of QTc prolongation varied across tricyclic antidepressants, with clomipramine and doxepin conferring the highest risk. Serotonin–norepinephrine reuptake inhibitors (eg, venlafaxine and duloxetine) were associated with increased risk of hyponatraemia and both gastrointestinal and sexual side-effects. Mirtazapine, a tetracyclic antidepressant, was associated with increased risk of sedation and weight gain, with an otherwise benign side-effect profile.

In the umbrella review of antipsychotic and antidepressant efficacy, of 295 citations retrieved, five meta-analyses met inclusion criteria. Two remained after selecting those with the largest numbers of drugs.^7^, ^8^ Confidence ratings for both meta-analyses were high.

The online tool, Psymatik Treatment Optimizer, is accessed via a dedicated website. The user interface of the Antipsychotic Treatment Optimizer is shown in the appendix (p 15); the Antidepressant Treatment Optimizer has the same format. The user is presented with a side-effect menu, with no limit on the number of side-effects that can be chosen. Side-effect concern ratings are defined using a slider, ranging between 0 (minor concern) and 100 (major concern). Concern ratings are synthesised with the effect size magnitudes in the side-effect databases using the TOPSIS method, ultimately providing a league table which ranks treatments from best to worst. Up to 10 993 pairwise drug–side-effect comparisons can be computed (if a user were to select all 14 side-effects for antipsychotics [5142 pairwise comparisons] and all nine side-effects for antidepressants [5851 pairwise comparisons]). The appendix (p 16) shows an example heatmap output of the Psymatik Antipsychotic Treatment Optimizer. In this example, the user has specified that they wish to avoid three side-effects: weight gain (85% concern), Parkinsonism (50% concern), and anticholinergic side-effects (15% concern). The rows of the heatmap correspond to individual drugs. The first heatmap column (from the left) corresponds to the overall TOPSIS side-effect scores that are specific to the user's concerns; the magnitude of these overall scores dictates the order of the drugs, with drugs ranked highest (best) placed at the top. The adjacent heatmap columns correspond to individual unweighted effect size magnitudes for the side-effects selected by the user. A separate column to the right of the heatmap provides efficacy data for each medication.

## Discussion

To our knowledge, we have compiled the most comprehensive side-effect databases for antipsychotics and antidepressants to date, incorporating meta-analytic data for more than 65 000 people, alongside a synthesis of seven national or international guidelines, for 69 drugs. Furthermore, for the first time to our knowledge, meta-analytic data were complemented by imputed results that were based on ordinal ranking scores, providing a compromise between the superior accuracy of meta-analytic results and broader drug coverage of guidelines.

Although the databases could be used in isolation to inform prescribing decisions, clinicians would still be faced with the challenge of simultaneously considering multiple side-effects for multiple drugs while also incorporating individual patient concerns. Our umbrella review highlighted the dilemma that often confronts clinicians and patients when making prescription decisions, whereby avoidance of one group of side-effects increases the risk of others (eg, for antipsychotics, choosing drugs with relatively low risk of weight gain and sedation results in the use of drugs with relatively high risk of movement side-effects and hyperprolactinaemia). The Psymatik Treatment Optimizer aids in these challenges by facilitating evidence-based, personalised, and comprehensive antipsychotic and antidepressant prescribing decisions that are based on drug–side-effect profiles. Although an online tool exists that uses a traffic light system to rank medications on the basis of anticholinergic side-effects,^48^ to our knowledge, the Psymatik Treatment Optimizer is the first application to address the issue of multidimensionality when simultaneously considering heterogenous side-effect profiles of antipsychotics and antidepressants along with patient preference. The power of Psymatik to allow comprehensive comparative assessments of drug side-effects is unparalleled; on the basis of the current databases, Psymatik computes up to 10 993 pairwise drug–side-effect comparisons in a fraction of a second. If 10 s of discussion were devoted to each of these comparisons, longer than 30 h would be required. Psymatik translates these comparisons into a visual output that is easy to understand for both patients and prescribers, providing an intuitive, scalable tool that can be used in evidence-based and shared decision making. In the UK, National Institute for Health and Care Excellence (NICE) guidelines for the management of schizophrenia do not specify which antipsychotics should be prescribed (except for clozapine in the setting of treatment resistance).^19^ Instead, it is stated that antipsychotic choice should follow a discussion of “the likely benefits and side-effects of each drug, including metabolic…extrapyramidal…cardiovascular…[and] hormonal”.^19^ Similar guidance is provided for treatment of depression, with NICE stating that before starting an antidepressant, a discussion should “cover the possible side-effects…including any side-effects [the patient] would particularly like to avoid”.^20^ The American Psychiatric Association guidelines also emphasise the importance of discussing side-effects as part of antipsychotic and antidepressant prescription decisions.^21^, ^22^ As such, the Psymatik Treatment Optimizer supports care delivery that is consistent with regulatory guidance for the treatment of schizophrenia and depression.^19^, ^20^, ^21^, ^22^

The Psymatik Treatment Optimizer is not intended to dictate prescribing decisions directly, but to facilitate informed discussions between the patient and prescriber about appropriate treatment. As such, this application holds promise as a tool for shared decision making within psychiatry. Shared decision making describes the collaborative process that involves a person and their health-care professional working together to reach a joint decision about care, whether that care is required immediately or in the future.^49^ NICE states that the delivery of shared decision making within health-care services should be embedded at an organisational level, and that as part of this process, treatment decision aids be employed.^49^ NICE also states that decision aids should be based on treatment information derived from “reliable, high-quality sources”, and that the aids be “quality assured” and linked to a “maintained database or signposted to those produced by national bodies”.^49^ The Psymatik Treatment Optimizer satisfies these requirements, and it is thus well positioned to facilitate gold-standard patient-centred care.

The premature mortality of people with serious mental illness is well recognised, with side-effects of treatments having a role.^50^ In the 2019 *Lancet Psychiatry* Commission for protecting the physical health of people with mental illness, reducing long-term side-effects of psychotropic medication was highlighted as a key future strategy for the field.^50^ Thus, by weighing up the relative side-effect burden of different psychotropics and facilitating the selection of treatments with comparatively benign side-effect profiles, the Psymatik Treatment Optimizer addresses a previously unmet clinical need.

Our study also has limitations. Our literature review highlighted evidence gaps that should be addressed by future studies. For example, although we identified comprehensive meta-analytic data for several antipsychotic side-effects, all of these side-effects required imputation to some extent, and data for three side-effects were based entirely on ordinal rankings. Furthermore, for several side-effects (eg, risk of hypertension and risk of venous thromboembolism) and for some antipsychotics or antidepressants, no data were available. Our search yielded a single meta-analysis examining antidepressant side-effects, which was not included in the database.^43^ This outcome was due to the modest number of included drugs in the meta-analysis, and concerns regarding the validity of meta-analytic and ordinal regression models, thereby precluding imputation. Specifically, we observed negative relationships between meta-analytic effect sizes and ordinal ranking scores for the two types of side-effects with sufficient data (insomnia or agitation; and nausea and vomiting). Of note, the meta-analysis in question examined maintenance studies.^43^ Symptoms such as insomnia and nausea and vomiting are typically considered to be acute antidepressant side-effects that can reduce with time;^30^ as such, they might be less prevalent in maintenance studies. Ordinal ranking scores might instead concern risk of acute side-effects, thereby explaining the regression model results. However, inadequate guideline referencing meant that we were unable to confirm this. Indeed, some guidelines did not provide any references to justify drug rankings. Furthermore, the quality of evidence on which rankings were based was invariably not reported. As such, although we were reassured by our assessments of guideline methodological quality, the accuracy and validity of guideline-derived data remain uncertain. Moreover, guidelines often used limited risk categories (eg, mild, moderate, and severe), meaning differentiation between some drugs for particular side-effects was not possible. To address concerns about data validity, accuracy, and granularity, future studies should aim to meta-analyse the side-effects for which we used ordinal ranking data. Unfortunately, any such future efforts are unlikely to solve the problem of absent side-effect data for older drugs, although the issue of absent data limits any approach to the delivery of evidence-based practice, not simply the approach by Psymatik. We also recognise that the risk and magnitude of some side-effects might be influenced by dose, and that use of higher doses in older trials might introduce risk of bias to some network meta-analysis results. Pragmatically, we focussed on side-effects that accompany large proportions of antipsychotics and antidepressants,^30^ but in doing so we did not gather data on rarer but serious side-effects such as neuroleptic malignant syndrome and serotonin syndrome and the side-effects typically associated with clozapine. Furthermore, although Psymatik allows for personalisation according to individual preferences, the current version does not account for patient features such as age, race and ethnicity, and sex, which might predispose to an increased risk of one side-effect over another. The influence of medication dose, polypharmacy, or lifestyle factors (eg, smoking) also are not accounted for. We recognise that randomised controlled trial populations might not wholly represent real-world patients, and that people with a higher than average side-effect tolerance or treatment responsiveness might be over-represented, potentially limiting the translation of results to clinical practice. However, we focussed on meta-analysis results from trial data to reduce heterogeneity and provide accurate assessments of drug-specific side-effects. Additionally, by focusing on monotherapy, we were unable to comment on potential drug–drug interactions that might translate into synergistic or antagonistic adverse or efficacy effects. Nevertheless, even naturalistic studies do not cover these highly complex aspects adequately. These issues could theoretically be addressed and included in future Psymatik versions. We also recognise that making judgements about relative side-effect concern is limited when the individual has not experienced those side-effects; however, this is a limitation that affects any initiative to facilitate discussions about side-effects.

With the potential to facilitate decision making, the Psymatik Treatment Optimizer should be implemented and tested to establish to what degree the tool could form a relevant part of future psychiatric practice. However, the utility of Psymatik is reliant on database accuracy, and we have identified that meta-analytic side-effect data for many psychiatric treatments are either absent or insufficient, which needs to be addressed by future studies. The side-effect databases will be updated as new side-effect data become available, with meta-analytic data replacing ordinal data. In time, the periodically updated databases will be replaced by a cumulative real time meta-analysis, whereby eligible raw trial data are added whenever published to provide the most up-to-date evidence. Qualitative assessments of application functionality will also be sought from patients, carers, and prescribers to inform the design of future Psymatik iterations. For example, by working with people with lived experience, a patient-facing tool could be developed to be used in advance of a clinical discussion. Furthermore, the health-economic impact of Psymatik will be examined.

We have compiled the most comprehensive antipsychotic and antidepressant side-effect databases to date, and we have developed the Psymatik Treatment Optimizer to support evidence-based, personalised, and comprehensive prescribing decisions.

## Data sharing

The corresponding author can be contacted for any data that are not included in the Article or appendix. Data will be provided dependent on the type of research request.

## Declaration of interests

TP has participated in educational speaker meetings organised by Lundbeck, Otsuka, Sunovion, Janssen, Schwabe Pharma, ROVI Biotech, and Recordati. RAMcC has participated in advisory or speaker meetings organised by Otsuka, Karuna, Boehringer Ingelheim, and Janssen. TP and RAMcC are directors of Pharmatik that funds website hosting for Psymatik. Neither TP nor RAMcC have holdings or financial stakes in any pharmaceutical company. MH has received honoraria for advisory boards and lectures from Recordati. SJ has participated in educational speaker meetings organised by Lundbeck, Otsuka, Sunovion, Janssen, and Boehringer Ingelheim. FG has received honoraria from Lundbeck, Otsuka, Sunovion, and Boehringer Ingelheim. ODH has received investigator-initiated research funding from or participated in advisory or speaker meetings organised by Angellini, Autifony, Biogen, Boehringer Ingelheim, Eli Lilly, Heptares, Global Medical Education, Invicro, Janssen, Lundbeck, Neurocrine, Otsuka, Sunovion, Rand, Recordati, Roche, Viatris (formerly Mylan), and ROVI Biotech. AHY has delivered paid lectures and advisory boards for the following companies: AstraZeneca, Boehringer Ingelheim, Eli Lilly, LivaNova, Lundbeck, Sunovion, Servier, Livanova, Janssen, Allegan, Bionomics, Sumitomo Dainippon Pharma, COMPASS Pathways, Sage, Novartis, and Neurocentrx. CUC has been a consultant or advisor to or has received honoraria from AbbVie, Acadia, Alkermes, Allergan, Angelini, Aristo, Boehringer Ingelheim, Cardio Diagnostics, Cerevel, CNX Therapeutics, COMPASS Pathways, Darnitsa, Gedeon Richter, Hikma, Holmusk, IntraCellular Therapies, Janssen, Johnson & Johnson, Karuna, LB Pharma, Lundbeck, MedAvante-ProPhase, MedInCell, Merck, Mindpax, Mitsubishi Tanabe Pharma, Mylan, Neurocrine, Newron, Noven, Novo Nordisk, Otsuka, Pharmabrain, PPD Biotech, Recordati, Relmada, Reviva, Rovi, Seqirus, SK Life Science, Sunovion, Sun Pharma, Supernus, Takeda, Teva, and Viatris; has provided expert testimony for Janssen and Otsuka; has served on a data safety monitoring board for COMPASS Pathways, Lundbeck, Relmada, Reviva, Rovi, Supernus, and Teva; has received grant support from Janssen and Takeda; has received royalties from UpToDate; and is a stock option holder of Cardio Diagnostics, MindPax, LB Pharma, and Quantic. All other authors declare no competing interests.
