# Supplementary material for: Antidepressant and antipsychotic side-effects and personalised prescribing: a systematic review and digital tool development
Source: Lancet Psychiatry. 2023 Nov;10(11):860–76. doi: 10.1016/S2215-0366(23)00262-6 (PMC10878984; doi:10.1016/S2215-0366(23)00262-6)
Supplement: Supplementary appendix [file mmc1.pdf]

THE LANCET
Psychiatry

Supplementary appendix

This appendix formed part of the original submission and has been peer reviewed. We post it as supplied by the authors.

Supplement to: Pillinger T, Howes OD, Correll CU, et al. Antidepressant and antipsychotic side-effects and personalised prescribing: a systematic review and digital tool development. *Lancet Psychiatry* 2023; published online Sept 26. https://doi.org/10.1016/S2215-0366(23)00262-6.

Antidepressant and antipsychotic side-effects and personalised prescribing: a systematic review and digital tool development

Toby Pillinger, Oliver D Howes, Christoph U Correll, Stefan Leucht, Maximillian Huhn, Johannes Schneider-Thoma, Fiona Gaughran, Sameer Jauhar, Philip K McGuire, David M Taylor, Allan H Young, Robert A McCutcheon

Appendix

Table of contents

PROSPERO Protocol………………………………………………………………………………………. 2
PRISMA Checklist……………………………………………..………………………………………... 6
Supplementary methods…………………………………………………………………………………. 9
Supplementary results…………………………………………………………………………………. 11
Supplementary references…………………………………………………………………………… 18

PROSPERO Protocol

Quantifying the side effect burden of antipsychotics and antidepressants: an umbrella review

Toby Pillinger, Robert McCutcheon

Citation

Toby Pillinger, Robert McCutcheon. Quantifying the side effect burden of antipsychotics and antidepressants: an umbrella review. PROSPERO 2022 CRD42022372142 Available from: https://www.crd.york.ac.uk/prospero/display_record.php?ID=CRD42022372142

Review question

What is the relative risk or magnitude of various side effects for the respective treatment of schizophrenia and major depressive disorder with antipsychotics and antidepressants?

Searches

We will search EMBASE, PsycINFO, and MEDLINE from inception to date the protocol is approved. Only meta-analyses written in English that are examining clinical trials will be selected.

Types of study to be included

Meta-analyses based on randomised controlled trials.

Condition or domain being studied

Schizophrenia and major depressive disorder.

Participants/population

People with schizophrenia or major depressive disorder.

Intervention(s), exposure(s)

Antipsychotic or antidepressant treatment (monotherapy).

Comparator(s)/control

Placebo.

Main outcome(s)

Antidepressant and antipsychotic side effects (as per Taylor DM, Barnes TRE, Young AH. The Maudsley Prescribing Guidelines in Psychiatry. Wiley Blackwell, 2021), as follows: extrapyramidal, parkinsonism, dyskinesia, dystonia, akathisia,

prolactin, headache, agitation, insomnia, cholinergic, gastrointestinal, constipation, nausea, arrythmia, QTc, hypotension, hypertension, weight, glucose, lipid, cholesterol, triglyceride, sedation, hyponatraemia, thromboembolism, sexual.

Measures of effect

As appropriate, relative risk or mean/standardised mean change in a given side effect.

Additional outcome(s)

None.

Data extraction (selection and coding)

Data will be extracted as follows: name of first author, year of publication, medication used in study, duration of study, as appropriate relative risk or mean/standardised mean change in a given side effect.

Risk of bias (quality) assessment

We are not planning on performing our own risk of bias assessments, as this is a review of meta-analyses. However, we will discuss the risk of bias assessments performed by those individual meta-analyses included in the umbrella review.

Strategy for data synthesis

The overarching goal is to create a summary database where relative side effect burden for antipsychotics and antidepressants is presented.

From each meta-analysis that our systematic review identifies that meets inclusion criteria, we will extract effect size magnitude for each side effect for each drug. Depending on the side effect, that effect size may be relative risk (e.g., risk of akathisia) or mean/standardised mean difference for a change in a given parameter (e.g., weight change). Where there is more than one meta-analysis for a side effect, we will select the meta-analysis with the largest sample size.

To allow readers to appreciate the relative risk or magnitude of side effects associated with each drug in a quantitative fashion, a heat map will be created. To do so, each effect effect size magnitude will be normalised (minimum-maximum scaled by subtracting the minimum value for that side effect and dividing by the range) to give values between 0 and 1. These normalised values will populate the heat map (rows = individual drugs, columns = individual side effects). This process will be performed in the programming language r.

The heat map / ranking diagram will used as the basis for us to formally discuss the relative side effect profiles of the drugs.

Analysis of subgroups or subsets

None planned.

Contact details for further information

Toby Pillinger
toby.pillinger@kcl.ac.uk

Organisational affiliation of the review

King's College London

Review team members and their organisational affiliations

Dr Toby Pillinger. King's College London
Dr Robert McCutcheon.

Type and method of review

Review of reviews, Systematic review

Anticipated or actual start date

07 November 2022

Anticipated completion date

06 March 2023

Funding sources/sponsors

National Institute for Health Research (NIHR)

Conflicts of interest

Language

English

Country

England

Stage of review

Review Ongoing

Subject index terms status

Subject indexing assigned by CRD

Subject index terms

Antidepressive Agents; Antipsychotic Agents; Depressive Disorder, Major; Humans; Risk; Schizophrenia

Date of registration in PROSPERO

04 November 2022

Date of first submission

01 November 2022

Stage of review at time of this submission

The review has not started

Stage	Started
Preliminary searches	No
Piloting of the study selection process	No
Formal screening of search results against eligibility criteria	No
Data extraction	No
Risk of bias (quality) assessment	No
Data analysis	No

The record owner confirms that the information they have supplied for this submission is accurate and complete and they understand that deliberate provision of inaccurate information or omission of data may be construed as scientific misconduct.

The record owner confirms that they will update the status of the review when it is completed and will add publication details in due course.

Versions

04 November 2022

eTable 1: PRISMA Checklist

Section and Topic	Item #	Checklist item	Location where item is reported
**TITLE**			
Title	1	Identify the report as a systematic review.	p1
**ABSTRACT**			
Abstract	2	See the PRISMA 2020 for Abstracts checklist.	p2
**INTRODUCTION**			
Rationale	3	Describe the rationale for the review in the context of existing knowledge.	pp4-5
Objectives	4	Provide an explicit statement of the objective(s) or question(s) the review addresses.	p5
**METHODS**			
Eligibility criteria	5	Specify the inclusion and exclusion criteria for the review and how studies were grouped for the syntheses.	p7 and Appendix p9
Information sources	6	Specify all databases, registers, websites, organisations, reference lists and other sources searched or consulted to identify studies. Specify the date when each source was last searched or consulted.	p6
Search strategy	7	Present the full search strategies for all databases, registers and websites, including any filters and limits used.	p6
Selection process	8	Specify the methods used to decide whether a study met the inclusion criteria of the review, including how many reviewers screened each record and each report retrieved, whether they worked independently, and if applicable, details of automation tools used in the process.	p7
Data collection process	9	Specify the methods used to collect data from reports, including how many reviewers collected data from each report, whether they worked independently, any processes for obtaining or confirming data from study investigators, and if applicable, details of automation tools used in the process.	p7
Data items	10a	List and define all outcomes for which data were sought. Specify whether all results that were compatible with each outcome domain in each study were sought (e.g. for all measures, time points, analyses), and if not, the methods used to decide which results to collect.	p7
	10b	List and define all other variables for which data were sought (e.g. participant and intervention characteristics, funding sources). Describe any assumptions made about any missing or unclear information.	n/a
Study risk of bias assessment	11	Specify the methods used to assess risk of bias in the included studies, including details of the tool(s) used, how many reviewers assessed each study and whether they worked independently, and if applicable, details of automation tools used in the process.	n/a, but quality assessments pp6-7
Effect measures	12	Specify for each outcome the effect measure(s) (e.g. risk ratio, mean difference) used in the synthesis or presentation of results.	p7
Synthesis methods	13a	Describe the processes used to decide which studies were eligible for each synthesis (e.g. tabulating the study intervention characteristics and comparing against the planned groups for each synthesis (item #5)).	p7
	13b	Describe any methods required to prepare the data for presentation or synthesis, such as handling of missing summary statistics, or data conversions.	p7
	13c	Describe any methods used to tabulate or visually display results of individual studies and syntheses.	p7

eTable 1: PRISMA Checklist

Section and Topic	Item #	Checklist item	Location where item is reported
	13d	Describe any methods used to synthesize results and provide a rationale for the choice(s). If meta-analysis was performed, describe the model(s), method(s) to identify the presence and extent of statistical heterogeneity, and software package(s) used.	p8
	13e	Describe any methods used to explore possible causes of heterogeneity among study results (e.g. subgroup analysis, meta-regression).	n/a
	13f	Describe any sensitivity analyses conducted to assess robustness of the synthesized results.	n/a
Reporting bias assessment	14	Describe any methods used to assess risk of bias due to missing results in a synthesis (arising from reporting biases).	n/a
Certainty assessment	15	Describe any methods used to assess certainty (or confidence) in the body of evidence for an outcome.	n/a
**RESULTS**			
Study selection	16a	Describe the results of the search and selection process, from the number of records identified in the search to the number of studies included in the review, ideally using a flow diagram.	Figure 1
	16b	Cite studies that might appear to meet the inclusion criteria, but which were excluded, and explain why they were excluded.	n/a
Study characteristics	17	Cite each included study and present its characteristics.	Table 1
Risk of bias in studies	18	Present assessments of risk of bias for each included study.	n/a, but AMSTAR in Table 1
Results of individual studies	19	For all outcomes, present, for each study: (a) summary statistics for each group (where appropriate) and (b) an effect estimate and its precision (e.g. confidence/credible interval), ideally using structured tables or plots.	n/a
Results of syntheses	20a	For each synthesis, briefly summarise the characteristics and risk of bias among contributing studies.	n/a
	20b	Present results of all statistical syntheses conducted. If meta-analysis was done, present for each the summary estimate and its precision (e.g. confidence/credible interval) and measures of statistical heterogeneity. If comparing groups, describe the direction of the effect.	n/a
	20c	Present results of all investigations of possible causes of heterogeneity among study results.	n/a
	20d	Present results of all sensitivity analyses conducted to assess the robustness of the synthesized results.	n/a
Reporting biases	21	Present assessments of risk of bias due to missing results (arising from reporting biases) for each synthesis assessed.	n/a
Certainty of evidence	22	Present assessments of certainty (or confidence) in the body of evidence for each outcome assessed.	n/a
**DISCUSSION**			
Discussion	23a	Provide a general interpretation of the results in the context of other evidence.	p11
	23b	Discuss any limitations of the evidence included in the review.	p13-14
	23c	Discuss any limitations of the review processes used.	p13-14
	23d	Discuss implications of the results for practice, policy, and future research.	pp14-15
**OTHER INFORMATION**			
Registration and protocol	24a	Provide registration information for the review, including register name and registration number, or state that the review was not registered.	p6

eTable 1: PRISMA Checklist

Section and Topic	Item #	Checklist item	Location where item is reported
	24b	Indicate where the review protocol can be accessed, or state that a protocol was not prepared.	p6
	24c	Describe and explain any amendments to information provided at registration or in the protocol.	n/a
Support	25	Describe sources of financial or non-financial support for the review, and the role of the funders or sponsors in the review.	pp15-16
Competing interests	26	Declare any competing interests of review authors.	p16
Availability of data, code and other materials	27	Report which of the following are publicly available and where they can be found: template data collection forms; data extracted from included studies; data used for all analyses; analytic code; any other materials used in the review.	p10 (psymatik link)

Supplementary Methods

Inclusion and exclusion criteria for meta-analyses
We included meta-analyses of randomised controlled trials examining monotherapy of either antidepressants or antipsychotics in the respective treatment of schizophrenia or major depressive disorder. To be included, drugs assessed within the meta-analysis needed a common comparator. We did not include meta-analyses of psychiatric disorders in the context of a physical health condition (e.g., post-stroke depression). To reduced heterogeneity, we did not include meta-analyses specifically examining children/adolescents/older age populations.

Selection of national/international guidelines for review
National/international guidelines or consensus statements for treatment of depression[1-11] and schizophrenia[1,12-18] were selected in line with previous reviews.[19,20]

Antipsychotic and antidepressant efficacy review
During the manuscript's review process, we worked to include efficacy data as part of Psymatik's output. As such, we conducted a second umbrella review which was not specified in the a priori protocol. We searched EMBASE, PsycINFO, and MEDLINE from inception to June week 3 2023 for meta-analyses that rank antidepressants or antipsychotics based on efficacy in adults with depression or schizophrenia respectively. The following search terms were used: '(antipsychotic or antidepressant) and efficacy and network meta-analysis'. To reduce heterogeneity, we only included meta-analyses of randomized controlled trials examining acute antidepressant or antipsychotic monotherapy in the respective treatment of major depressive disorder or schizophrenia in adults. Methodological quality of each meta-analysis was assessed using the 'A Measurement Tool to Assess Systematic Reviews' (AMSTAR) 2 checklist; confidence in meta-analytic results was categorized as high, moderate, low, or critically low. To create the efficacy database, meta-analytic effect sizes for overall symptom improvement were extracted separately by TP and RM and cross-checked with differences resolved by discussion.

Quality assessment of meta-analyses
Methodological quality of each meta-analysis was assessed using the 'A Measurement Tool to Assess Systematic Reviews' (AMSTAR) 2 checklist.[21] Rating overall confidence was as specifed by AMSTAR2 as follows:[21] High (no or one non-critical weakness); Moderate (more that one non-critical weakness); Low (one critical flaw with or without non-critical weaknesses); Critically Low (more than one critical flaw with or without non-critical weaknesses).

Quality assessment of guidelines
We assessed methodological quality of Guidelines using the Appraisal Guideline Research and Evaluation Europe (AGREE) II reporting checklist.[22] The AGREE II instrument assesses both the quality of reporting and the quality of the guideline development process. It provides an appraisal of the predicted validity of a guideline, which is the likelihood that it will achieve its intended outcome. The AGREE instrument consists of 23 key items grouped into six quality domains with a seven-point Likert scale to score each item. The six domains are:

1. Scope and purpose (3items)
2. Stakeholder involvement (3 items)
3. Rigour of development (8 items)
4. Clarity and presentation (3 items)
5. Applicability (4 items)
6. Editorial independence (2 items)

We summed the scores of the individual items within a domain and the whole six domains and standardised the total as a percentage of the maximum possible score. We defined overall score quality as per the following thresholds: x ≥80%, outstanding; 80%> x ≥70%, excellent; 70%> x ≥60%, good; 60%> x ≥50%, satisfactory; 50%> x ≥40%, poor; x < 40%, very poor.

Explanation of side-effect database creation
To create the side-effect database, meta-analytic effect sizes were extracted in duplicate by TP and RM. If 'mean difference' and 'standardised mean difference' were both provided for a given side-effect, the analysis with superior assessments of heterogeneity was selected. Ordinal data from guidelines ranking drugs based on

magnitude/risk of specific side-effects were also extracted. Both meta-analytic and ordinal outcomes were normalised (minimum-maximum scaled by subtracting the minimum value for that side-effect and dividing by the range) to provide values between 0 and 1. Where there were multiple guidelines ranking data for the same side-effect, a mean of normalised scores was calculated. Meta-analytic data were preferred over ordinal data. Where there was more than one meta-analysis for a given side-effect, we selected the meta-analysis examining the largest number of drugs. All antipsychotic/antidepressant formulations were considered, where meta-analyses presented results for different formulations of the same drug (e.g., oral and long-acting injectable), we selected the result with the largest sample size. We imputed missing meta-analytic data based on ordinal ranking scores, as follows. Where there were ≥10 overlapping data points, linear regression was used to define the relationship between ordinal ranking scores (predictor/independent variable) and meta-analytic effect sizes (predicted/dependent variable) for a given side-effect; this model was then used to predict missing meta-analytic effect sizes based on available ranking scores. Where there were multiple meta-analyses examining the same side-effect for the same number of drugs, we selected the meta-analysis with the largest regression model coefficient of determination. Although meta-analyses are typically considered to be at the top of the 'evidence pyramid',[23] the validity of some side-effect NMA results have been queried, with inclusion of small outlier studies blamed.[24] Therefore, we excluded meta-analytic effect sizes for drug side-effects where there were major uncertainty concerns. Major uncertainty for a given drug side-effect was defined as an accompanying 95% confidence interval that was at least double the mean of 95% confidence intervals for all drugs for that side-effect. Excluded meta-analytic results were replaced by imputed data. To minimise exclusion of drugs, results (either direct or imputed) from meta-analyses were only used if available for ≥66% available drugs. Where this was not possible, ordinal ranking data were added instead. The resultant side-effect database consisted of rows corresponding to drugs, and columns corresponding to side-effects, with entries corresponding to meta-analytic effect sizes, imputed meta-analytic effect sizes, or guideline-defined ordinal ranking scores.

Supplementary Results

Rationale for antipsychotic side-effect meta-analytic selection

Huhn et al., 2019[24] provided the largest meta-analyses for the following side-effects: change in weight, risk of Parkinsonism, risk of akathisia, QTc prolongation, risk of sedation, and risk of anticholinergic side-effects. Similarly, the largest NMAs examining risk of tardive dyskinesia (Carbon et al., 2018),[25] change in total cholesterol (Burschinski et al., 2023)[26] and prolactin (Zhu et al., 2021)[27] were selected. For change in glucose and triglycerides, two NMAs were identified examining the same number: Pillinger et al., 2020[28] and Burschinski et al., 2023.[26] For both these parameters, the meta-analyses from Pillinger et al., 2020[28] had the largest ordinal/meta-analytic regression model coefficients of determination, and were therefore selected for data extraction (Pillinger glucose model R^2=0.24, Burschinksi glucose model R^2=0.11; Pillinger triglyceride model R^2=0.44, Burschinski triglyceride model R^2=0.06).Imputation of meta-analytic data for total cholesterol and triglyceride changes were based on ordinal ranking scores for 'risk of dyslipidaemia'. Although 'dyslipidaemia' broadly describes raised cholesterol and triglyceride levels,[29] the term can also describe various alterations in subtypes of cholesterol.[29] Since it was unclear which cholesterol subtypes the ordinal data were referring to, we chose not to use these data to impute meta-analytic values for LDL and HDL cholesterol. Moreover, owing to insufficient drug numbers, meta-analytic data were not extracted for the following side effects: insomnia, agitation/anxiety, hypersalivation, and change in body mass index. Since we had already extracted meta-analytic data for risk of both akathisia and parkinsonism, we did not extract data for risk of 'extra-pyramidal side-effects'. Furthermore, although ordinal data were available for risk of galactorrhoea, dysmenorrhoea, constipation, and neuroleptic malignant syndrome, drug coverage for these side-effects did not meet the threshold for database inclusion.

eTable 2. Guideline Selection

Guideline reviewed	Ordinal ranking data provided?
The Maudsley Prescribing Guidelines in Psychiatry[1]	Yes
NICE. Depression in adults: treatment and management: [2]	No
Guideline Development Panel for the Treatment of Depressive D. Summary of the clinical practice guideline for the treatment of depression across three age cohorts[3]	No
Evidence-based guidelines for treating depressive disorders with antidepressants: A revision of the 2008 British Association for Psychopharmacology guidelines[4]	Yes
Canadian Network for Mood and Anxiety Treatments (CANMAT) 2016 Clinical Guidelines for the Management of Adults with Major Depressive Disorder[5,6]	No
Ministry of Health Clinical Practice Guidelines: Depression. [7]	No
Royal Australian and New Zealand College of Psychiatrists clinical practice guidelines for mood disorders. [8]	No
Pharmacological treatment of unipolar depressive disorders: summary of WFSBP guidelines[9]	No
Practice Guideline for the Treatment of Patients With Major Depressive Disorder[10]	No
UpToDate, Wolters Kluwer[11]	Yes
Psychosis and schizophrenia in adults: prevention and management[12]	No
Evidence-based guidelines for the pharmacological treatment of schizophrenia: recommendations from the British Association for Psychopharmacology[13]	No
International Psychopharmacology Algorithm Project (www.ipap.org): Schizophrenia Algorithm Flowchart[14]	No
Ministry of Health Clinical Practice Guidelines: Schizophrenia[15]	No
Royal Australian and New Zealand College of Psychiatrists clinical practice guidelines for the treatment of schizophrenia and related disorders[16]	Yes
World Federation of Societies of Biological Psychiatry (WFSBP) - Guidelines for biological treatment of schizophrenia[17]	Yes
The American Psychiatric Association Practice Guideline for the Treatment of Patients With Schizophrenia[18]	Yes

eTable 3. Ordinal/meta-analytic regression models for antipsychotic side-effects

	R2	coef	Se
sedation	0.374	1.3464	0.363
anticholinergic_effects	0.389	1.5749	0.403
parkinsonism	0.552	2.8433	0.546
akathisia_and_agitation	0.341	7.7583	2.201
weight_gain	0.649	0.6070	0.108
prolactin_and_sexual_side_effects	0.703	1.8376	0.290
QTc	0.411	0.5772	0.219
hyperglycaemia	0.236	0.3508	0.182
tc	0.376	0.2850	0.095
hyperlipidemia	0.251	0.6664	0.347
tardive_dyskinesia	0.637	5.9032	1.485

eTable 4. Data sources for antipsychotic side-effects

Side-effect	Meta-analysis	Imputed (absent meta-analytic data)	Imputed (uncertain meta-analytic data)	Ordinal ranking data	Data not available
**Parkinsonism**	29	0	3	NA	0
**Akathisia**	31	0	1	NA	0
**Dystonia**	0	0	0	22	10
**TD**	12	10	2	NA	8
**PRL**	21	7	1	NA	3
**QTc**	12	13	2	NA	5
**Anticholinergic**	30	0	1	NA	1
**Sedation**	29	0	2	NA	1
**Weight gain**	23	5	3	NA	1
**Triglycerides**	14	12	0	NA	6
**Cholesterol**	16	7	1	NA	7
**Glucose**	15	10	0	NA	7
**Hypotension**	0	0	0	27	5
**Seizure**	0	0	0	24	8

Cell numbers correspond to number of antipsychotics

eTable 5. Guideline methodological quality results

Guideline	Scope & Purpose	Stakeholder Involvement	Rigour of Development	Clarity of presentation	Applicability	Editorial independence	Total Score
**Schizophrenia**							
American Psychiatric Association[18]	100%	100%	95%	100%	36%	93%	87% Outstanding
The Maudsley Prescribing Guidelines in Psychiatry[1]	100%	66%	50%	95%	21%	64%	61% Good
Royal Australian and New Zealand College of Psychiatrists[16]	100%	100%	50%	100%	32%	93%	70% Excellent
World Federation of Societies of Biological Psychiatry[17]	100%	66%	38%	100%	21%	0%	52% Satisfactory
**Depression**							
British Association for Psychopharmacology[4]	100%	100%	73%	100%	36%	93%	79% Excellent
The Maudsley Prescribing Guidelines in Psychiatry[1]	100%	66%	50%	95%	21%	64%	61% Good
Wolters Kluwer UpToDate[11]	100%	66%	34%	100%	32%	21%	54% Satisfactory

eTable 6. Ordinal/meta-analytic regression model for antidepressant side-effects

	R2	Coef	SE
Nausea & vomiting	0.14	-0.26	0.20
Insomnia/agitation	0.38	-0.63	0.25

Based on meta-analytic data from Kishi et al., 2023[30]

Antipsychotic and antidepressant efficacy umbrella review

Of 295 citations retrieved, 5 meta-analyses met inclusion criteria. Two studies remained after selecting those with the largest sample sizes.[24,31] Confidence ratings for both meta-analyses were ‘high’.

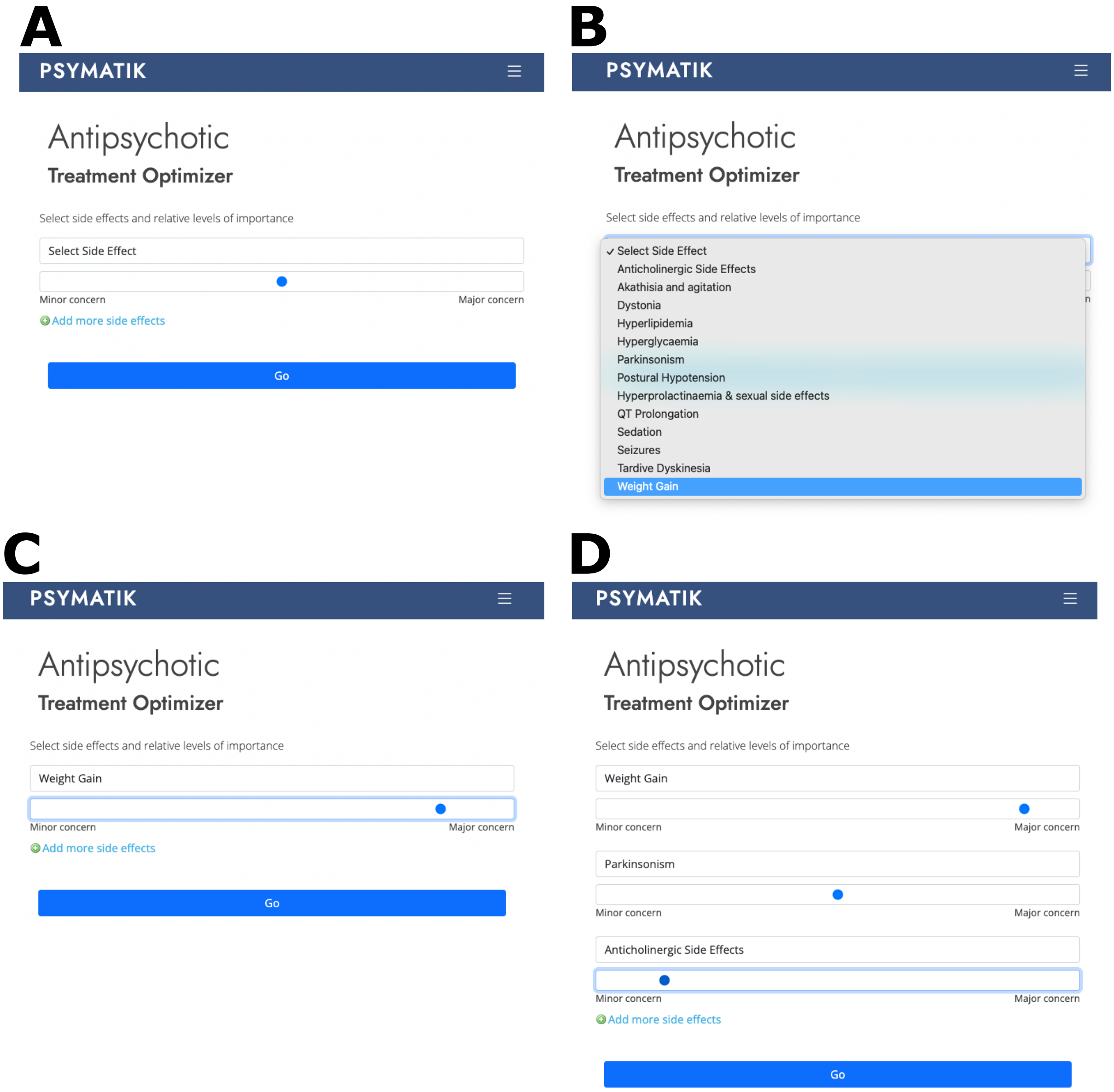

Figure. The Psymatik Antipsychotic Treatment Optimizer user interface
A, the Antipsychotic Treatment Optimizer home page; B, the user is presented with a side-effect menu with no limit on number of side-effects that can be chosen; C, the user defines the extent they wish to avoid a side-effect using a slider; D, a slider for each side-effect determines the relative extent the user wishes to avoid one side-effect compared to others.

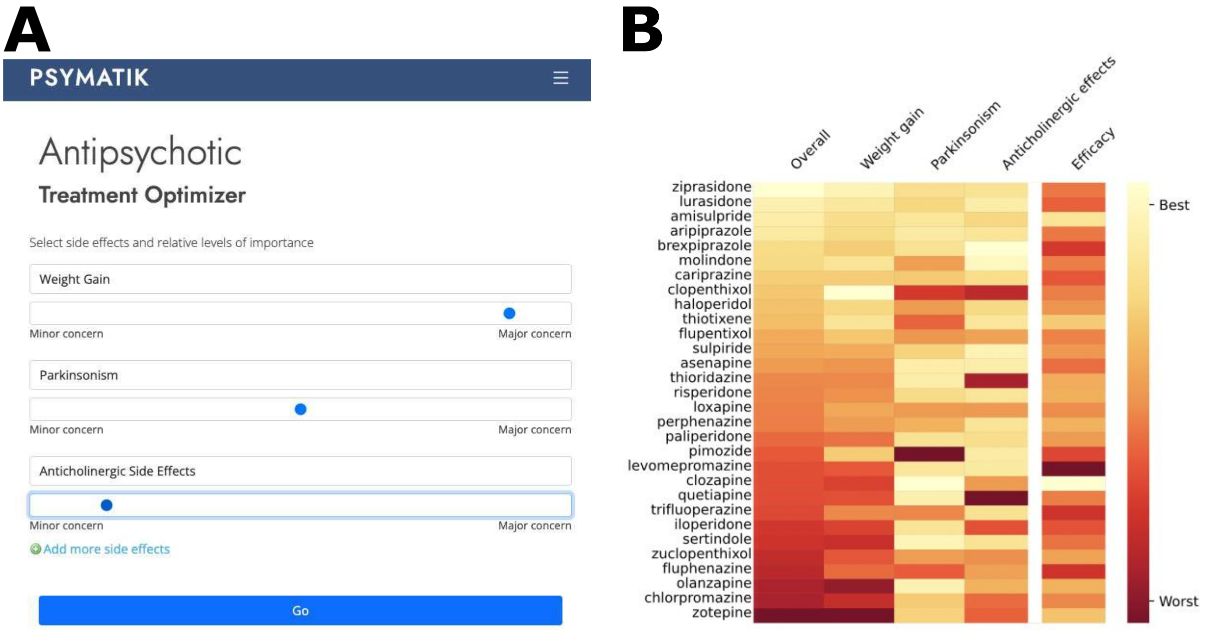

Figure. Psymatik Antipsychotic Treatment Optimizer output

Figure A shows the user has specified that they wish to avoid 3 side-effects: weight gain (85% concern); Parkinsonism (50% concern); and anticholinergic side-effects (15% concern). Figure B shows the Psymatik heatmap output. The rows of the heatmap correspond to individual drugs. The first heatmap column (from the left) corresponds to the overall (summated) Psymatik side-effect scores that is specific to the user's concerns; the magnitude of these overall scores dictates the order of the drugs, with drugs ranked highest (best) placed at the top. The subsequent heatmap columns correspond to individual unweighted effect size magnitudes for those side-effects selected by the user.

References

1. Taylor DM, Barnes TRE, Young AH. The Maudsley Prescribing Guidelines in Psychiatry: Wiley Blackwell; 2021.
2. NICE. Depression in adults: treatment and management. London; 2022.
3. Guideline Development Panel for the Treatment of Depressive D. Summary of the clinical practice guideline for the treatment of depression across three age cohorts. *Am Psychol* 2021.
4. Cleare A, Pariante CM, Young AH, et al. Evidence-based guidelines for treating depressive disorders with antidepressants: A revision of the 2008 British Association for Psychopharmacology guidelines. *Journal of Psychopharmacology* 2015; **29**(5): 459-525.
5. Kennedy SH, Lam RW, McIntyre RS, et al. Canadian Network for Mood and Anxiety Treatments (CANMAT) 2016 Clinical Guidelines for the Management of Adults with Major Depressive Disorder: Section 3. Pharmacological Treatments. *Can J Psychiatry* 2016; **61**(9): 540-60.
6. Milev RV, Giacobbe P, Kennedy SH, et al. Canadian Network for Mood and Anxiety Treatments (CANMAT) 2016 Clinical Guidelines for the Management of Adults with Major Depressive Disorder: Section 4. Neurostimulation Treatments. *Can J Psychiatry* 2016; **61**(9): 561-75.
7. Chua HC, Chan LL, Chee KS, et al. Ministry of Health Clinical Practice Guidelines: Depression. *Singapore Medical Journal* 2012; **53**(2): 137-43.
8. Malhi GS, Bassett D, Boyce P, et al. Royal Australian and New Zealand College of Psychiatrists clinical practice guidelines for mood disorders. *Aust N Z J Psychiatry* 2015; **49**(12): 1087-206.
9. Bauer M, Severus E, Moller HJ, Young AH, Disorders WTFoUD. Pharmacological treatment of unipolar depressive disorders: summary of WFSBP guidelines. *Int J Psychiatry Clin Pract* 2017; **21**(3): 166-76.
10. Gelenberg A, Freeman MP, Markowitz J, et al. Practice Guideline for the Treatment of Patients With Major Depressive Disorder (Third Edition). *American Psychiatric Association* 2010.
11. UpToDate. https://www.uptodate.com/ Wolters Kluwer, data accessed 15 Sept 2022
12. NICE. Psychosis and schizophrenia in adults: prevention and management. London; 2014.
13. Barnes TRE, Psychopharmacology BA. Evidence-based guidelines for the pharmacological treatment of schizophrenia: recommendations from the British Association for Psychopharmacology. *Journal of Psychopharmacology* 2011; **25**(5): 567-620.
14. IPAP. International Psychopharmacology Algorithm Project (www.ipap.org): Schizophrenia Algorithm Flowchart. 2019.
15. Verma S, Chan LL, Chee KS, et al. Ministry of Health Clinical Practice Guidelines: Schizophrenia. *Singapore Medical Journal* 2011; **52**(7): 521-5.
16. McGorry P, Killackey E, Lambert T, et al. Royal Australian and New Zealand College of Psychiatrists clinical practice guidelines for the treatment of schizophrenia and related disorders. *Australian and New Zealand Journal of Psychiatry* 2005; **39**(1-2): 1-30.
17. Falkai P, Wobrock T, Lieberman J, et al. World Federation of Societies of Biological Psychiatry (WFSBP) - Guidelines for biological treatment of schizophrenia, part 1: Acute treatment of schizophrenia. *World Journal of Biological Psychiatry* 2005; **6**(3): 132-91.

18. APA. The American Psychiatric Association Practice Guideline for the Treatment of Patients With Schizophrenia. Third Edition. 2020.
19. Howes OD, Thase ME, Pillinger T. Treatment resistance in psychiatry: state of the art and new directions. *Mol Psychiatry* 2022; **27**(1): 58-72.
20. Howes OD, McCutcheon R, Agid O, et al. Treatment-Resistant Schizophrenia: Treatment Response and Resistance in Psychosis (TRRIP) Working Group Consensus Guidelines on Diagnosis and Terminology. *Am J Psychiatry* 2017; **174**(3): 216-29.
21. Shea BJ, Reeves BC, Wells G, et al. AMSTAR 2: a critical appraisal tool for systematic reviews that include randomised or non-randomised studies of healthcare interventions, or both. *BMJ* 2017; **358**: j4008.
22. Brouwers MC, Kerkvliet K, Spithoff K, Consortium ANS. The AGREE Reporting Checklist: a tool to improve reporting of clinical practice guidelines. *BMJ* 2016; **352**: i1152.
23. Berlin JA, Golub RM. Meta-analysis as evidence: building a better pyramid. *JAMA* 2014; **312**(6): 603-5.
24. Huhn M, Nikolakopoulou A, Schneider-Thoma J, et al. Comparative efficacy and tolerability of 32 oral antipsychotics for the acute treatment of adults with multi-episode schizophrenia: a systematic review and network meta-analysis. *Lancet* 2019; **394**(10202): 939-51.
25. Carbon M, Kane JM, Leucht S, Correll CU. Tardive dyskinesia risk with first- and second-generation antipsychotics in comparative randomized controlled trials: a meta-analysis. *World Psychiatry* 2018; **17**(3): 330-40.
26. Burschinski A, Schneider-Thoma J, Chiocchia V, et al. Metabolic side effects in persons with schizophrenia during mid- to long-term treatment with antipsychotics: a network meta-analysis of randomized controlled trials. *World Psychiatry* 2023; **22**(1): 116-28.
27. Zhu Y, Zhang C, Siafis S, et al. Prolactin levels influenced by antipsychotic drugs in schizophrenia: A systematic review and network meta-analysis. *Schizophr Res* 2021; **237**: 20-5.
28. Pillinger T, McCutcheon RA, Vano L, et al. Comparative effects of 18 antipsychotics on metabolic function in patients with schizophrenia, predictors of metabolic dysregulation, and association with psychopathology: a systematic review and network meta-analysis. *Lancet Psychiatry* 2020; **7**(1): 64-77.
29. Fredrickson DS. An international classification of hyperlipidemias and hyperlipoproteinemias. *Ann Intern Med* 1971; **75**(3): 471-2.
30. Kishi T, Ikuta T, Sakuma K, et al. Antidepressants for the treatment of adults with major depressive disorder in the maintenance phase: a systematic review and network meta-analysis. *Mol Psychiatry* 2023; **28**(1): 402-9.
31. Cipriani A, Furukawa TA, Salanti G, et al. Comparative efficacy and acceptability of 21 antidepressant drugs for the acute treatment of adults with major depressive disorder: a systematic review and network meta-analysis. *Lancet* 2018; **391**(10128): 1357-66.
